# miR-194 inhibits the proliferation, invasion, migration, and enhances the chemosensitivity of non-small cell lung cancer cells by targeting forkhead box A1 protein

**DOI:** 10.18632/oncotarget.7545

**Published:** 2016-02-21

**Authors:** Xuchao Zhu, Dan Li, Fei Yu, Chengyou Jia, Jing Xie, Yushui Ma, Suyun Fan, Haidong Cai, Qiong Luo, Zhongwei Lv, Lihong Fan

**Affiliations:** ^1^ Department of Nuclear Medicine, Shanghai Tenth People's Hospital, Tongji University School of Medicine, Shanghai, PR China; ^2^ Department of Respiration, Shanghai Tenth People's Hospital, Tongji University School of Medicine, Shanghai, PR China

**Keywords:** miR-194, non-small cell lung cancer, forkhead box A1 protein

## Abstract

Recent studies have implied that miRNAs may play a crucial role in tumor progression and may be involved in the modulation of some drug resistance in cancer cells. Earlier studies have demonstrated that miR-194 was involved in tumor metastasis and drug resistance in non-small cell lung cancer (NSCLC), whereas their expression and roles on NSCLC still need further elucidation. In the current study, we found that miR-194 is decreased in NSCLC samples compared with adjacent non-cancerous lung samples, and low expression of miR-194 predicts poor patient survival. Both *in vitro* and *in vivo* experiments showed that ectopic stable expression miR-194 suppressed proliferation, migration, invasion and metastasis and induced apoptosis in NSCLC cells and that this suppression could be reversed by reintroducing forkhead box A1 (FOXA1), a functional target of miR-194. In addition, miR-194 was downregulated in in cisplatin-resisted human NSCLC cell line-A549/DDP and overexpression of miR-194 increases cisplatin sensitivity. These findings suggested that miR-194 inhibits proliferation and metastasis and reverses cisplatin-resistance of NSCLC cells and may be useful as a new potential therapeutic target for NSCLC.

## INTRODUCTION

Non-small cell lung cancer (NSCLC) is one of the most common cancers and one of the leading causes of cancer related mortality across the world [1]. Most common pulmonary malignancy are NSCLC, it accounts for 85% of cases of pulmonary malignancies [2]. The most effective treatment for NSCLC is surgical resection, but most of patients diagnosed with NSCLC have reached an advanced pathological stage, at which point the patients miss the best time for surgical resection. Despite significant advances in surgery, chemotherapy and radiotherapy, the 5-year survival rate for lung cancer was only 15%. Therefore, new treatment strategies to identify new molecular targets for prevention and treatment of NSCLC are urgently needed.

MicroRNAs (miRNAs) are a class of single-stranded noncoding RNA molecules of 18–24 nucleotides in length. They play a key role in many biological processes through negative regulation of the expression of target genes post-transcriptionally by sequence-specific binding to the 3′ untranslated regions (UTRs) of specific mRNA targets [3].

MiR-194 is a vertebrate specific microRNA with a known role in mitochondrial energy production [4], inhibition of inflammation [5], chondrogenesis [6] and neuronal differentiation [7]. It has also been implicated in a number of malignancies, including oral squamous cell carcinoma [8], breast cancer [9, 10] and so on. Another study showed that miR-194, via its actions on the protein FOXM1, inhibits gastric carcinoma cell migration, invasion, and epithelial to mesenchymal transition [11]. In oral squamous cell carcinoma, miR-194 appears to medicate PI3K-Akt-FOXO3a signaling pathway via its negative regulation of AGK [8]. A recent study reported that miR-194 was decreased and associated with tumor size and tumor differentiation in colorectal cancer, overexpression of miR-194 suppresses tumor growth by regulating the MAP4K4/c-Jun/MDM2 signaling pathway. MiR-194, commonly repressed in colorectal cancer, suppresses tumor growth by regulating the MAP4K4/c-Jun/MDM2 signaling pathway. Moreover, miR-194 was found to reduce in primary tumor samples with metastasis and enforcing miR-194 suppressed motility and invasiveness of lung cancer cells *in vitro* and *in vivo* through downregulation of two key functional factors, BMP1 and p27^kip1^. miR-194 suppresses metastasis of non-small cell lung cancer through regulating expression of BMP1 and p27^kip1^. However, the roles of miR-194 in NSCLC growth and metastasis and the molecular mechanism remain to be investigated.

FOXA1 is a member of the human Forkhead-box family. These genes have been implicated in congenital disorders, diabetes, and carcinogenesis [12]. In squamous cell carcinoma of the lung, FOXA1 expression has been shown to be associated with distant metastases and poorer overall survival [13]. It has also been shown to promote epithelial to mesenchymal transition (EMT) in NSCLC, perhaps explaining its association with the propensity to metastasize in NSCLC cells [14]. More recently, FOXA1 was found to upregulate and high FOXA1 expression have lower rates of progression free survival in Urothelial carcinoma of the bladder. MicroRNA-99a and 100 mediated upregulation of FOXA1 in bladder cancer.

In this study, we first determined the miR-194 expression in NSCLC tissues and their corresponding adjacent normal tissues, then investigated the functional role of miR-194 in tumourigenesis, metastasis, and apoptosis induction in NSCLC cells. We also provide experimental evidence that miR-194 regulated cellular function via directly interacting with the FOXA1 mRNA at the 3′-UTR. In all, our data supports the notion that miR-194 acts as a tumor suppressor and might be a novel potential therapeutic target for NSCLC.

## RESULTS

### miR-194 was significantly downregulated and correlated with poor prognosis

**Figure 1:**
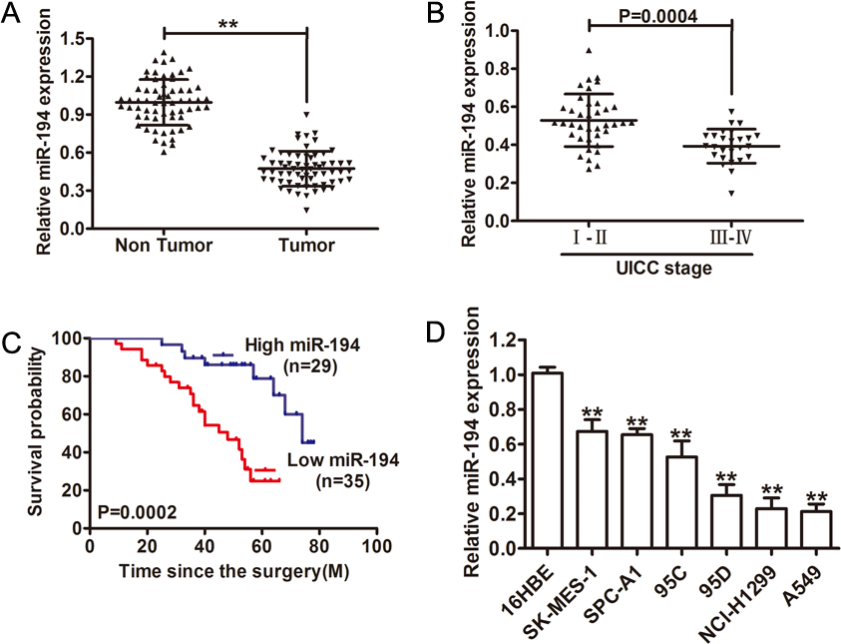
Relative miR-194 expression in NSCLC tissue and its clinical significance (**A**) Relative expression of miR-194 expression in NSCLC tissue (*n* = 64) and in paired adjacent non-cancerous tissues (*n* = 64). (**B**) Relative expression of miR-194 expression in NSCLC patients with stage I–II disease and with stage III–IV disease. (**C**) Kaplan-Meier analysis of overall survival in patients with tumors that express high levels of miR-194 versus patients with tumors that express low levels of miR-194. (**D**) The expression levels of miR-194 in multiple NSCLC cell lines relative to the benign 16HBE cell line were assessed by qRT-PCR. MiR-194 expression was normalized to U6 expression. **P < 0.01.

Expression levels of miR-194 were determined in 64 pairs of NSCLC tissue and paired adjacent non-tumor tissue. Expression of miR-194 in NSCLC tumor tissue was significantly lower than in the paired non-tumor tissue (*p* < 0.01) (Figure 1A). Expression of miR-194 was also examined in NSCLC tissues of varying stage. In higher stage lesions (stage III–IV), miR-194 expression was significantly lower than in lower stage lesions (stage I–II) (*p* = 0.0004) (Figure 1B). Furthermore, we investigated the potential associations between miR-194 expression and patients’ clinicopathological variables. Clinicopathological variables of NSCLC patients were shown in Table 1. Interestingly, low miR-194 expression was significantly correlated with Lymph node metastasis and TNM stage (*P* < 0.05). Overall survival was examined in patients with NSCLC's expressing varying amounts of miR-194. 29 patients had tumors that expressed high levels of miR-194, while 35 patients had tumors that expressed low levels of miR-194. Patients with tumors that expressed high levels of miR-194 had significantly longer overall survival than patients who had tumors that expressed low levels of miR-194 (*p* = 0.0002) (Figure 1C). Finally, expression levels of miR-194 were determined in six NSCLC cell lines, with the benign human bronchial epithelial cell line (16HBE) serving as a control. Expression levels of miR-194 were significantly less in all of the NSCLC cell lines compared to the control (*p* < 0.01) (Figure 1D), especially, NCI-H1299 and A549 cells showed lowest miR-194 levels. Overall, these results indicated that not only does decreased expression of miR-194 distinguish benign tissue from malignant NSCLC but also that the magnitude of the decrease in expression in tumor tissue can characterize the aggressiveness of the tumor.

### miR-194 inhibits NSCLC cell proliferation both *in vitro* and *in vivo*

**Figure 2:**
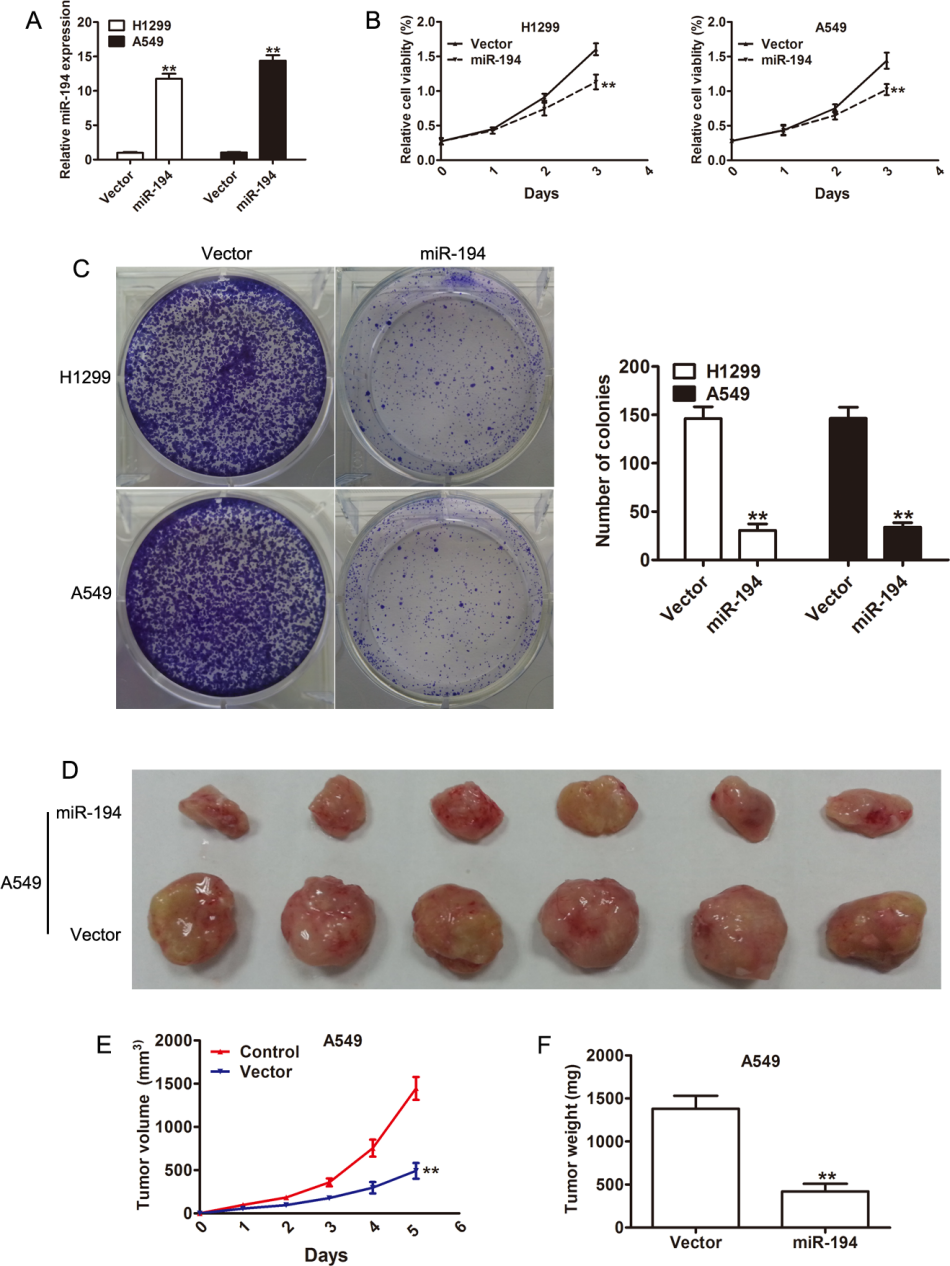
Overexpression of miR-194 suppresses NSCLC cell proliferation (**A**) Overxpression of miR-194 in H1299 and A549 cells transfected with the miR-194 vector with respect to cells transfected with the control vector was confirmed by qRT-PCR. (**B**) CCK-8 assays demonstrating the effect of miR-194 overexpression on the proliferation of H1299 and A549 NSCLC cells. (**C**) Colony formation assays demonstrating the effect of miR-194 overexpression on the proliferation of H1299 and A549 NSCLC cells. Error bars represent mean ± the standard deviation from three independent experiments. Representative colony formation images are also provided. (**D**) A photo of dissected tumors demonstrates the smaller size of tumors that were derived from A549 cells that overexpressed miR-194. (**E-F**) Tumor volume was measured weekly, and tumor weight was determined at day 35. **P < 0.01.

With the knowledge that lower level of miR-194 is associated with more aggressive NSCLC behavior, we then attempted to determine what factors contribute to the increase in aggressiveness of these tumors. We therefore examined effect of miR-194 on cell proliferation. Transfection of the H1299 and A549 NSCLC cell lines with either a miR-194 vector or an empty control vector was performed. Increased expression of miR-194 in the cells transfected with the miR-194 vector was confirmed by qRT-PCR in both cell lines (*p* < 0.01) (Figure 2A). The MTT assay was then performed on cells from both lines to assess cell viability. Both H1299 and A549 cells that were transfected with the miR-194 vector demonstrated significantly reduced cell viability compared to H1299 and A549 cells transfected with the control vector respectively after three days (*p* < 0.01) (Figure 2B). The colony formation assay was also performed to assess proliferative capacity. Again, both H1299 and A549 cells that were transfected with the miR-194 vector exhibited significantly lower rates of colony formation than H1299 and A549 cells transfected with the control vector respectively (*p* < 0.01). Representative micrographs of this assay are also provided (Figure 2C). Finally, *in vivo* analysis of tumor growth was assessed by measuring both tumor volume and weight. A549 cells that were transfected with the miR-194 vector and implanted into nude mice grew into both significantly smaller (*p* < 0.01) (Figure 2E) and lighter (*p* < 0.01) (Figure 2F) tumors than A549 cells that were transfected with the empty control vector and implanted into nude mice. Gross specimens dissected from the mice pictorially demonstrate this difference in volume (Figure 2D). Overall, these results suggested that miR- 194 has an inhibitory effect on tumor aggressiveness by reducing cell proliferation both *in vitro* and *in vivo*.

### miR-194 arrests the cell cycle and induces apoptosis in NSCLC cells

**Figure 3:**
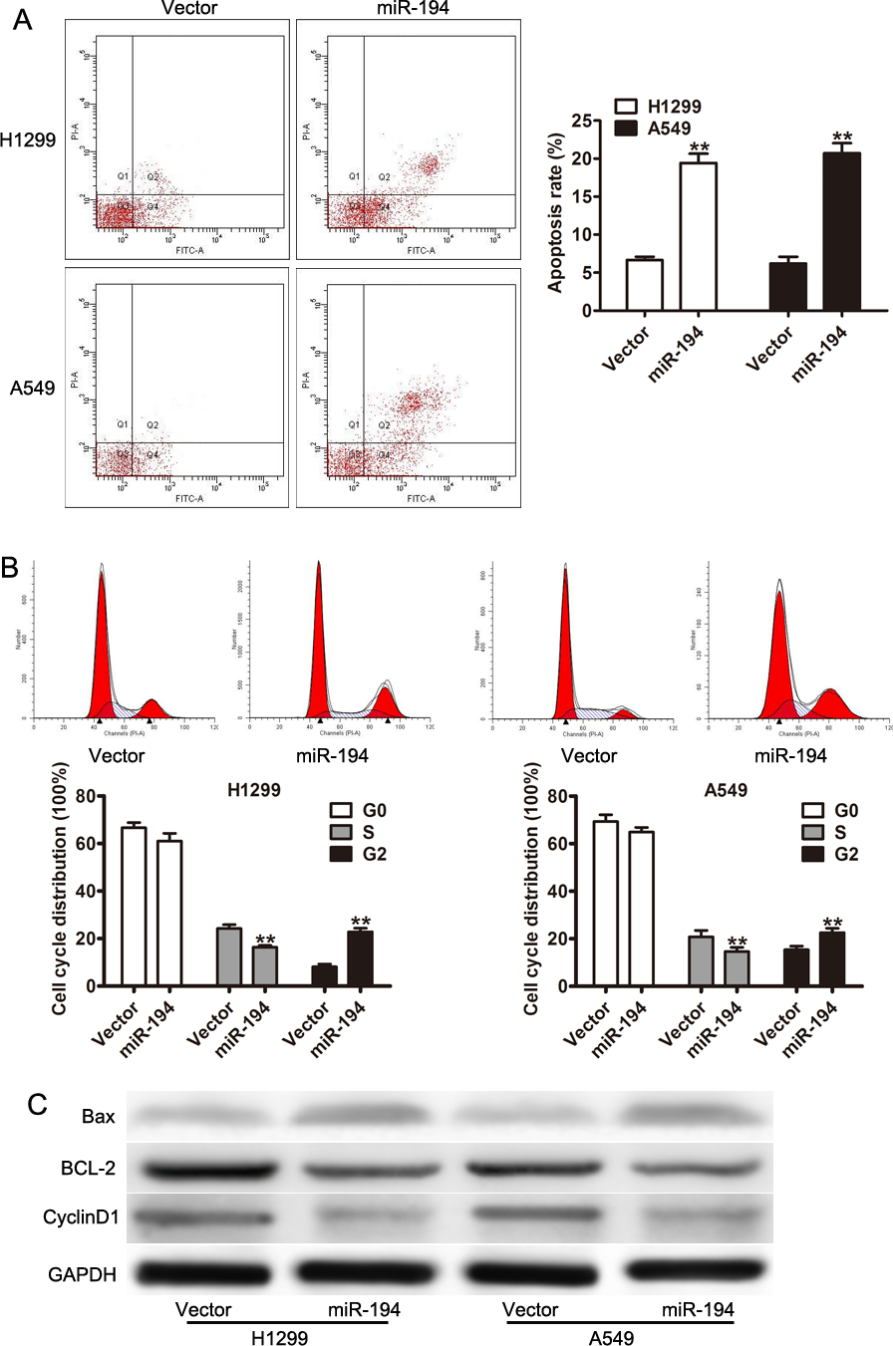
miR-194 arrests the cell cycle and induces apoptosis in NSCLC cells (**A**) Rates of apoptosis in H1299 and A549 cells transfected with the miR-194 vector versus the empty vector control. (**B**) Cell cycle assay demonstrating the percentage of H1299 and A549 cells overexpressing miR-194 in the G0, S, and G2 phases compared to control. (**C**) Western blot demonstrating expression levels of CyclinD1, BCL-2, and Bax in H1299 and A549 cells overexpressing miR-194 compared to control. GADPH was used as the loading control. **P < 0.01.

The reduction in cell proliferation seen in NSCLC cells treated with miR-194 was then further examined by looking at its effect on apoptosis and the cell cycle. The FITC and PI apoptosis assays were used to determine rates of apoptosis in the various cells. Both H1299 and A549 cells that were transfected with the miR-194 vector exhibited significantly increased rates of apoptosis compared to H1299 and A549 cells transfected with the control vector respectively (*p* < 0.01) (Figure 3A). A cell cycle assay was subsequently performed to determine the percent of cells in each phase of the cell cycle. A significantly lower percent of both H1299 and A549 cells transfected with the miR-194 vector were in S phase than H1299 and A549 cells transfected with the control vector respectively (*p* < 0.01) (Figure 3B). Conversely, a significantly higher percent of both H1299 and A549 cells transfected with the miR- 194 vector were in G2 phase than H1299 and A549 cells transfected with the control vector respectively (*p* < 0.01) (Figure 3B). These findings suggested that miR-194 reduces cellular proliferation in NSCLC tissue both by increasing apoptosis as well as by inhibiting cellular mitosis.

Finally, expression levels of specific proteins known to be involved in the regulation of apoptosis were determined by Western blot. Both H1299 and A549 cells that were transfected with the miR-194 vector expressed greater levels of Bax and lower levels of BCL-2 and CyclinD1 than H1299 and A549 cells transfected with the control vector respectively (Figure 3C). As Bax is a protein necessary for the initiation of apoptosis, its increased expression in cells treated with the miR- 194 vector confirms the association between miR-194 expression and apoptosis. Conversely, as BCL-2 inhibits apoptosis and Cyclin D1 promotes progression through the cell cycle, their reduced expression in cells treated with miR-194 vector also confirm the association between miR-194 expression and apoptosis.

### miR-194 inhibits NSCLC migration and invasion both *in vitro* and *in vivo*

**Figure 4:**
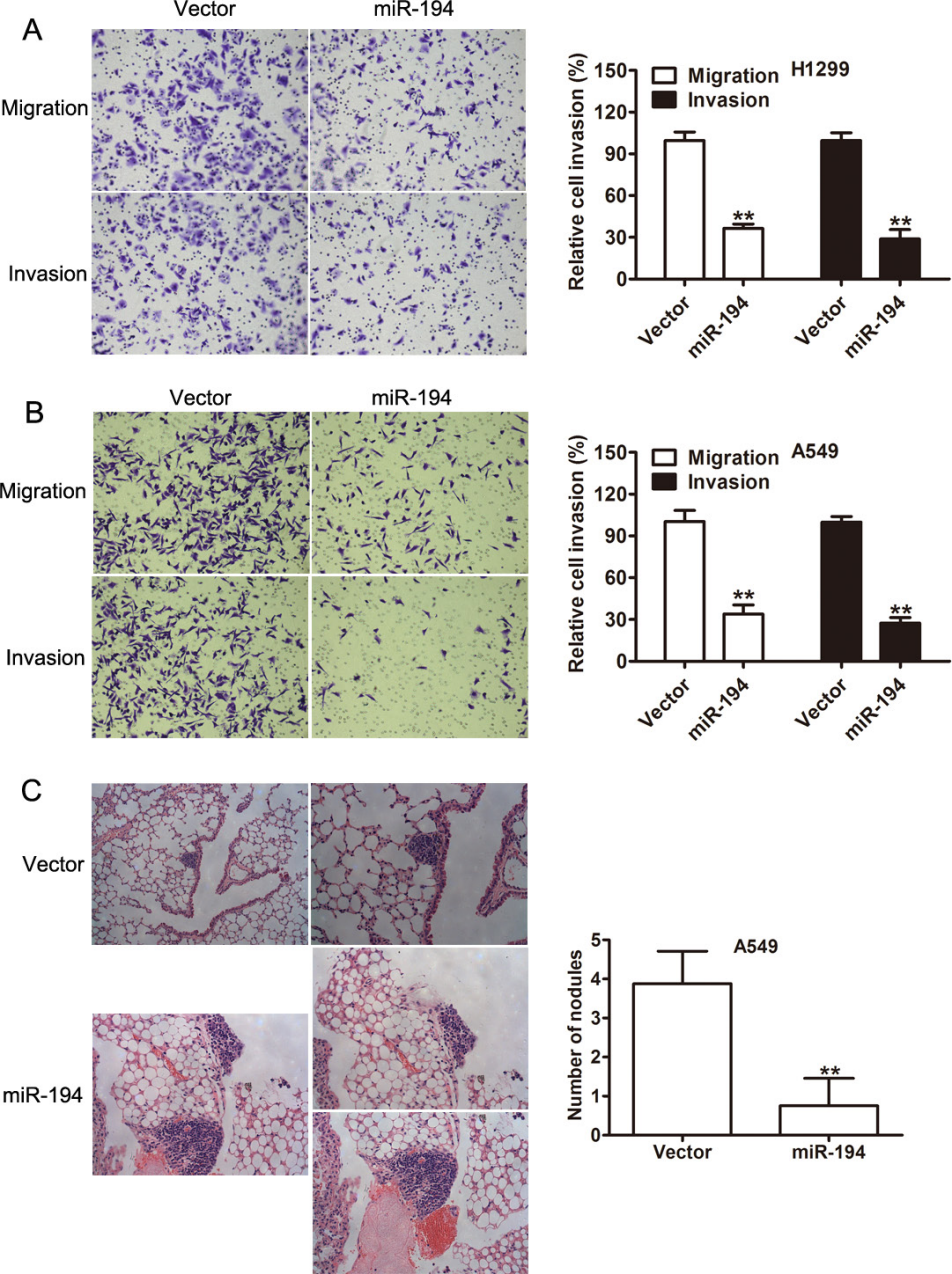
miR-194 inhibits the migratory, invasive, and metastatic capacities of NSCLC cells (**A–B**) Migration and invasion assays of H1299 and A549 NSCLC cells transfected with the control vector or the miR-194 vector. Representative micrographs demonstrating migrating and invasive cells are also provided. Average number of invasive or migratory cells per high power field from three independent experiments ± the standard deviation is shown at the right. (**C**) Mice injected with A549 cells transfected with the miR- 194 vector had significantly fewer pulmonary metastases than control. Representative histologic section from the dissected lungs of the mice are also provided. Magnification: ×100 (left), ×200 (right). **P < 0.01.

Cell proliferation is an important measure of tumor aggressiveness, though it is an incomplete measure. A tumor's ability to metastasize is a vital component of its aggressiveness and is directly related to its migratory and invasive capacity. Therefore, migration and invasion assays were performed. The numbers of invasive and migratory cells per high power field were counted. Both H1299 (*p* < 0.01) (Figure 4A) and A549 (*p* < 0.01) (Figure 4B) cells that were transfected with the miR-194 vector exhibited significantly reduced rates of migratory and invasive capacity compared to H1299 and A549 cells transfected with the control vector respectively.

Given that NSCLC cells treated with the miR-194 vector exhibited reduced migratory and invasive capacity *in vitro*, we therefore examined the metastatic capacity of these cells *in vivo*. Thin histologic sections of the lungs were obtained from mice that had been implanted with tumor cells. The number of metastatic nodules were counted. Mice that had been implanted with A549 cells treated with the miR-194 vector had a significantly lower number of pulmonary metastases than mice that had been implanted with A549 cells treated with the control vector (*p* < 0.01). Representative micrograph sections from the dissected lungs are shown (Figure 4C).

### FOXA1 is a direct target of miR-194

**Figure 5:**
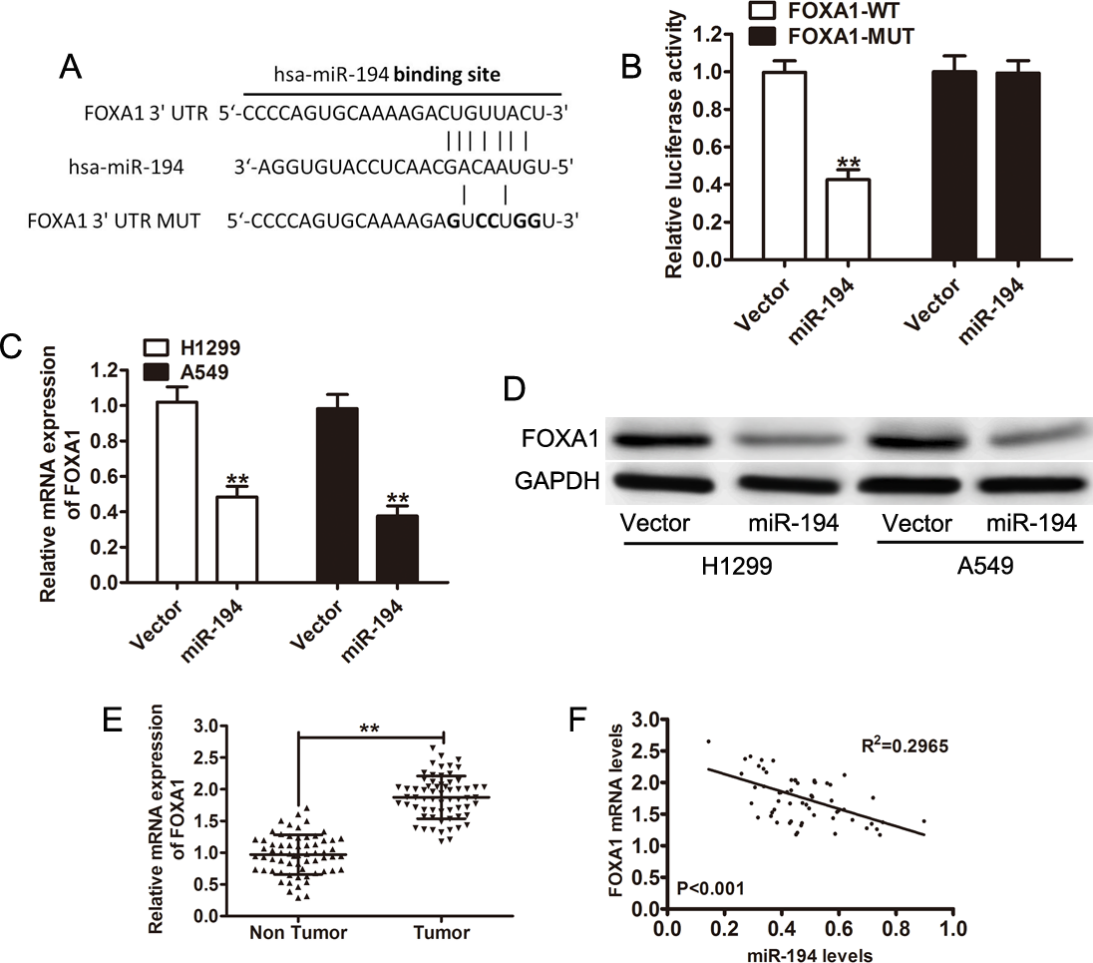
FOXA1 is a direct target of miR-194 (**A**) TargetScan predicts a putative binding site for miR-194 in the 3′-UTR of FOXA1. A mutated FOXA1 variant was also constructed. (**B**) A luciferase assay was performed on H1299 cells co-transfected with miR- 194 and reporter vectors carrying either a FOXA1 3′-UTR with a wild-type (FOXA1-WT) or mutated (FOXA1-MUT) miR-194 response element. (**C-D**) FOXA1 mRNA and protein expression levels in H1299 and A549 cells transfected with either empty vector or the miR-194 vector. (**E**) Relative FOXA1 mRNA levels in NSCLC tissues (*n* = 64) and in paired adjacent non-cancerous tissues (*n* = 64). (**F**) Pearson correlation analysis demonstrates an inverse relationship between FOXA1 mRNA levels and miR-194 levels. **P < 0.01.

Because miR-194 expression is associated with both decreased tumor growth and metastatic capacity, a search for a potential binding protein was performed. FOXA1 is one such protein, TargetScan demonstrated a putative binding site to miR-194 in the 3′ UTR region of FOXA1 (FOXA1-WT) (Figure 5A). A mutated FOXA1 construct (FOXA1-WT) was also created, with mutations at the binding site (Figure 5A). A luciferase assay was subsequently performed on H1299 cells transfected with either the miR-194 vector or the control vector and subsequently treated with either the FOXA1-WT protein or the FOXA1-MUT protein. Cells co-transfected with the miR-194 vector and the FOXA1-WT protein had significantly lower levels of luciferase activity than cells co-transfected with the control vector and the FOXA1-WT protein (*p* < 0.01) (Figure 5B). Conversely, cells co-transfected with the miR-194 vector and the FOXA1-MUT protein had similar levels of luciferase activity as cells co-transfected with the control vector and the FOXA1-MUT protein (Figure 5B).

Quantitative real-time PCR and Western blot were then used to determine expression levels of native FOXA1 in cells transfected with the miR-194 vector. Both H1299 (*p* < 0.01) and A549 (*p* < 0.01) cells that were transfected with the miR-194 vector had significantly lower levels of FOXA1 mRNA than H1299 and A549 cells transfected with the control vector respectively (Figure 5C). Subsequently, cells transfected with the miR-194 vector also had reduced expression of FOXA1 (Figure 5D). FOXA1 mRNA levels were also measured in non-tumor tissue. NSCLC tissue had significantly higher levels of FOXA1 mRNA than paired non-tumor tissue (*p* < 0.01) (Figure 5E). Further, Pearson correlation analysis demonstrated an inverse relationship between FOXA1 mRNA levels and miR-194 levels (*p* < 0.001) (Figure 5F). Overall, the above findings suggest that binding of miR- 194 to FOXA1 results in the degradation of the FOXA1 protein. When miR-194 levels are low, as in NSCLC tissue, FOXA1 levels remain elevated.

### Expression of FOXA1 could partially restore the pro-apoptotic and anti-invasion function of miR-194

**Figure 6:**
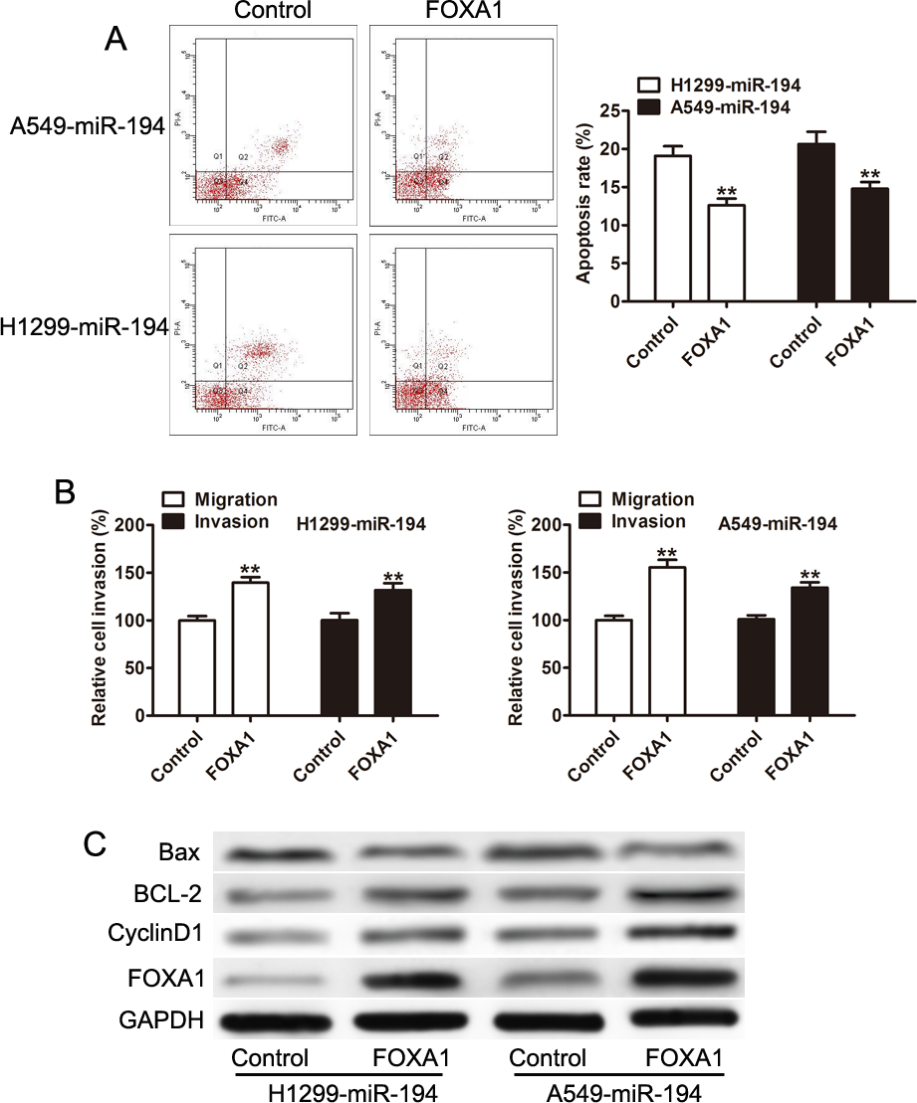
Overxpression of FOXA1 inhibits the pro-apoptotic, anti-invasive, and anti-migratory capacities attributed to miR-194 (**A**) Overexpression of FOXA1 reduces the rate of apoptosis in both H1299 and A549 cells treated with the miR-194 vector. (**B**) Overexpression of FOXA1 promotes the migratory and invasive capacities of H1299 and A549 cells treated with miR-194. (**C**) Western blot demonstrating expression levels of FOXA1, CyclinD1, BCL-2, and Bax in H1299 and A549 cells transfected with the miR-194 vector. Overexpression of FOXA1 results in an anti-apoptotic expression pattern compared to control. **P < 0.01.

Having confirmed that miR-194 acts to reduce FOXA1 levels in NSCLC cells, we then wished to determine whether this action of miR-194 is responsible for its tumorigenic effects. We therefore reintroduced FOXA1 expression in NSCLC cells that overexpressed miR-194 and determined its effect on apoptosis and metastatic capacity. Overexpression of FOXA1 significantly reduced the rates of apoptosis in NSCLC cells. Both H1299-miR-194 (*p* < 0.01) and A549-miR-194 (*p* < 0.01) cells that were induced to overexpress FOXA1 demonstrated reduced rates of apoptosis compared to the control (Figure 6A). Overexpression of FOXA1 also restored both the migratory and invasive capacities of NSCLC cells transfected with miR-194. Both H1299-miR-194 (*p* < 0.01) and A549-miR-194 (*p* < 0.01) cells that were induced to overexpress FOXA1 exhibited significantly increased capacities to both migrate and invade compared to the control (Figure 6B). Finally, western blot was used to confirm anti-apoptotic action of FOXA1. Both H1299-miR-194 and A549-miR-194 cells overexpressing FOXA1 expressed greater levels of the anti-apoptotic proteins BCL-2 and Cyclin D1 and recued levels of the pro-apoptotic protein Bax compared to control (Figure 6C). These findings suggest that FOXA1 is responsible for the tumorigenic effects in NSCLC cells, and reduction of FOXA1 via miR-194 inhibits those effects.

### miR-194 regulates the sensitivity of NSCLC cells to DDP *in vitro*

**Figure 7:**
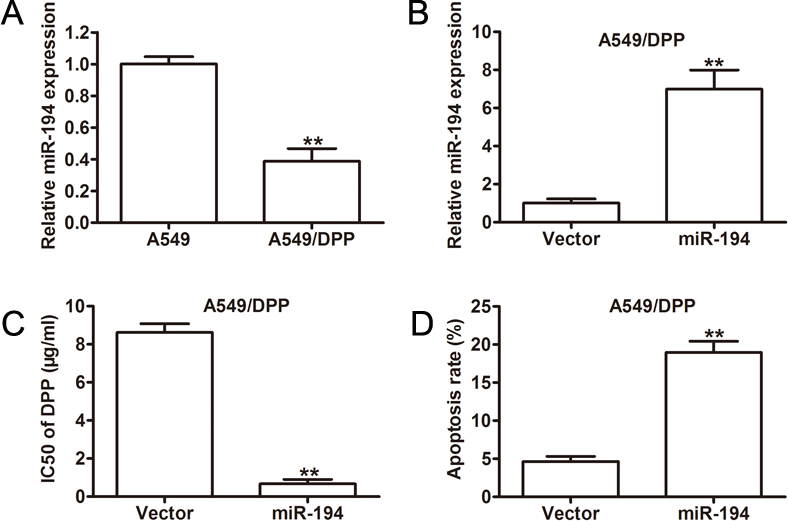
Overexpression of miR-194 in A549/DPP cells significantly increases their sensitivity to DPP (**A**) Relative miR-194 expression in A549 parent cells and A549/DPP cells. (**B**) qRT-PCR analysis of miR-194 expression in A549/DPP cells transfected with either empty vector or the miR-194 vector. (**C**) IC50 values for the anticancer drug DPP in A549/DPP cells transfected with either empty vector or the miR-194 vector. (**D**) Rates of apoptosis in A549/DPP cells transfected with either empty vector or the miR-194 vector as determined by flow cytometry.

Finally, we wished to assess the sensitivity of NSCLC cells to chemotherapy based on their expression level of miR-194. We first examined miR-194 expression in cisplatin-resistant NSCLC cells (A549/DPP). A549/DPP cells expressed significantly lower levels of miR- 194 than A549 cisplatin-sensitive cells (*p* < 0.01) (Figure 7A). We then examined miR-194 expression in A549/DPP cells transfected with the miR-194 vector versus the control vector. These cells expressed significantly greater levels of miR-194 than the control (*p* < 0.01) (Figure 7B), thus confirming the efficacy of the vector in the cisplatin-resistant line. Next, we examined the half maximal inhibitory concentration of DPP for A549/DPP cells transfected with the miR-194 vector. These cells had a significantly lower half maximal inhibitory concentration (IC50) than cells transfected with the control vector (*p* < 0.01) (Figure 7C). Lastly, we determined the rate of apoptosis in A549/DPP cells transfected with the miR- 194 vector. These cells exhibited a significantly higher rate of apoptosis than the control (*p* < 0.01) (Figure 7D). Overall, these findings suggest that increased expression of miR-194 improves the sensitivity of NSCLC cells to the chemotherapeutic agent DPP, whereby cells that express higher levels of miR-194 not only have higher rates of apoptosis but also require a significantly smaller amount of the drug to produce the same effect.

## DISCUSSION

Pathologically, miR-194 may play a role in the development of osteoporosis [15, 16] and osseous developmental abnormalities [17]. It has also been implicated in a number of malignancies, including bladder cancer [18], gastric cancer [11, 19, 20], endometrial cancer [21, 22], renal cell carcinoma [23], prostate cancer [24], pancreatic adenocarcinoma [25, 26], osteosarcoma [27], colorectal carcinoma [28, 29], multiple myeloma [30], hepatocellular carcinoma (HCC) [30], and esophageal adenocarcinoma [31, 32]. Additionally, miR-194 may play an indirect role in the prevention of HCC, it has been shown to regulate the hepatitis C virus binding target CD81, thereby preventing entry of the virus into hepatocytes [33].

In this study, we have demonstrated a mechanism that miR-194 was decreased in clinical NSCLC tissues compared to adjacent normal lung tissues and that miR-194 played a critical role in NSCLC progression by regulating cell proliferation and invasion via negatively regulate FOXA1. The roles of miR-194 in this study were consistent with other studies, which showed significant growth inhibition in several cancer cell types [34–37]. In gastric cancer, overexpression of miR-194 was associated with decreased tumor size and less advanced tumor stage, though was not associated with cell proliferation [19]. Yet another study examining miR-194's role in preventing the progression of gastric cancer suggested that it was miR-194's regulation of the protein RBX1 that was responsible for its inhibitory effect. In endometrial cancer, miR-194 was also found to have a protective effect, increasing levels of E-cadherin and reducing levels of vimentin, thus inhibiting cellular invasion via its regulation of the protein BMI-1 [21]. Patients with endometrial cancers that produced higher levels of miR-194 also had a better prognosis [22]. In renal cell carcinoma (RCC), miR- 194 overexpression results in reduced migratory and invasive capacity of RCC cells [23]. In osteosarcoma, overexpression of miR-194 inhibits tumor growth and metastatic potential via the regulation of CDH2 and IGF1R [27]. In colorectal carcinoma, miR-194 inhibits cell viability and invasion via its actions on the AKT2 pathway. It is also expressed in lower amounts with increasing tumor grade [28]. Another study had similar findings regarding the role of miR-194 in colorectal carcinoma, though it suggested that miR-194 regulated the MAP4K4/c-Jun/MDM2 signaling pathway [29]. In my present study, overexpression of miR-194 inhibited cell proliferation, migration, and invasion *in vitro* and *in vivo*. These data consisted with previous results that miR-194 inhibits the capacity of NSCLC cells to metastasis [30]. And our data also showed that miR-194 was decreased in NSCLC tissues and associated with poor prognosis.

Cisplatin resistance is still a major obstacle for the clinical therapy of NSCLC with advanced stage. Our data showed that miR-194 was significantly decreased in stage III–IV. It is interesting to investigate whether miR-194 would affect the NSCLC cells sensitive to DDP. As we expected, overexpression of miR-194 are more prone to apoptosis when treated with a DDP and require a significantly reduced half maximal inhibitory concentration of the chemotherapeutic agent. Our findings suggest that miR-194 could play an important role in the development of cisplatin resistance in NSCLC, consisted with the results miR-194 was reduced in A549/DDP. (Alterations of microRNAs in cisplatin-resistant human non-small cell lung cancer cells (A549/DDP)).

In summary, we showed that low levels of miR-194 are associated with worse prognosis of NSCLC and could inhibit cell proliferation and invasion *in vitro* and *in vivo*. Furthermore, a Together, our findings suggest that miR-194 acts as a tumor-inhibiting factor in NSCLC, and could be a target for NSCLC, even patients with chemotherapy tolerance.

## MATERIALS AND METHODS

### Patients and tissue samples

Paired NSCLC tissue and adjacent normal lung tissue samples were obtained from 64 patients who had undergone surgical resection without prior radiotherapy or chemotherapy between April 2008 and May 2012 at the department of Respiration of Shanghai 10th People's Hospital, affiliated to Tongji University in Shanghai, China. Histopathologic, diagnostic, and clinicopathologic staging of the patients from which the samples were obtained was performed according to the current International Union Against Cancer (UICC) protocol. The use of these tissue specimens was approved by the ethics committee of Shanghai 10th People's Hospital and conducted in adherence with the Declaration of Helsinki protocols. Written informed consent was obtained from all patients. Specimens were frozen in liquid nitrogen and stored at −80°C for further use.

### Cell culture

Six NSCLC cell lines (A549, SPC-A1, 95C, 95D, NCI-H1299 and SK-MES-1) and a normal human bronchial epithelial cell line (16HBE) were purchased from the Institute of Biochemistry and Cell Biology of the Chinese Academy of Sciences (Shanghai, China) and cultured in DMEM supplemented with 10% fetal bovine serum (FBS) (Invitrogen, Carlsbad, USA), 100 units/ml penicillin and 100 μg/ml streptomycin (Hyclone, Logan, USA) in a humidified incubator at 37°C with 5% CO_2_. A cisplatin-resistant human non-small cell lung cancer cell line (A549/DPP) was established and conserved by our lab laboratory and maintained in a1 μg/l final concentration of cisplatin as previously described [9].

### RNA extraction and quantitative real-time PCR

Total RNA was extracted from cells using TRIzol reagent (Invitrogen) according to the manufacturer's protocol. For FOXA1 expression, reverse transcription was performed with Superscript first-strand synthesis system (Invitrogen) according to the manufacturer's instruction. The following primers were used: FOXA1 forward, 5′- GC AATACTCGCCTTACGGCT-3′ and reverse, 5′- TACA CACCTTGGTAGTACGCC-3′; GAPDH forward, 5′-ACA ACTTTGGTATCGTGGAAGG-3′ and reverse 5′-GCC ATCACGCCACAGTTTC-3′. GAPDH was used as a loading control. To quantify miR-194, the expression level of miR-194 was measured by the TaqMan miRNA Assay (ABI) according to the manufacturer's protocol. U6 snRNA were used as an internal loading control. qRT-PCR was performed on an ABI 7900HT instrument (Applied Biosystems, USA). All reactions were run in triplicate and all experiments were carried at least three independent times.

### Plasmid construction and lentivirus production

An overexpression of miR-194 vector was created according to our previous publications [9] with following primer 5′-AAAGGATCCCAGGAGTTGTAAA TCCGAGCCG-3′ and the reverse primer 5′-AAAGAATTC TTCATAGGTCAGAGCCCTGTGCA-3′. A full length of human FOXA1 3′-UTR with a wide type and mutant target sequence for miR-194 were cloned into downstream of the of Renilla psi-CHECK2 (Promega, Madison, WI, USA) according to our previous publications. To force expression of FOXA1, DNA fragment coding FOXA1 protein was amplified by PCR and then subcloned into the pcDNA3.1 vector (Clontech, Palo Alto, CA, USA) with primers: Forward: 5′- AAAGCTAGCATGTTAGGA ACTGTGAAGATGGAAG-3′; Reverse: 5′-AAACTCGA GCTAGGAAGTGTTTAGGACGGGTCT-3′. All plasmids were confirmed by DNA sequencing.

The lentiviruses of miR-194 were generated by co-transfecting HEK293T with pPACKH1 Lentivector Packaging KIT (SBI) according to our previous publications [9]. To generate the stable cell line, cells were transfected with lentiviruses for 24 h and replaced with complete culture medium. Infection efficiency was confirmed by qRT-PCR 96 h after infection.

### Luciferase reporter assay

The luciferase reporter gene vector with wild type FOXA1 (FOXA1-WT) or mutate FOXA1 (FOXA1-MUT) co-transfected with miR-194 in HEK293T cells. Cells were washed 48 h after transfection, lysed and assayed using Dual-Luciferase Reporter Assay reagent (Promega, Madison, USA) according to the manufacturer's instructions.

### Cell viability, colony-formation, cell-cycle, and apoptosis assays

Cell viability was evaluated using the Cell Counting Kit 8 (CCK8, Dojindo, Japan) described in our previous publications [9]. In brief, cells were seeded in 96-well plates at a density of 1 × 10^3^ cells/well in 100-μl medium. 100 μ CCK-8was added to the culture at 1, 2, 3 days. Then the OD value was measured at 450 nm in a spectrophotometer (Spectra Max Plus, Molecular Devices, Sunnyvale, USA).

Cells were seeded into 6-well plates as 800 cells/well for 14 days. Then, the medium was refreshed with medium containing 10% FBS once every 3 days, and the colony formation was observed. Colonies with more than 40 cells were counted. Four fields were employed, and the mean was obtained. In addition, crystal violet staining (Shanghai Jingtian Biotech Co., Ltd., JT-00518) was applied and representative photographs were captured.

Apoptotic cells were examined using an Annexin V-PE Apoptosis Detection Kit (Millipore, USA). The samples were harvested and then stained with 5 μl of annexin V-PE and 5 μl of PI. Flow cytometer was performed to detect cellular apoptosis, and the results were analyzed using the Cell-QuestTM Pro software (BD Biosciences, USA). Pretreated cells were harvested, fixed in ice-cold 70% ethanol at 4°C overnight, and then resuspended in 1 mL of PBS containing 1 mg/mL RNase (Sigma, St. Louis, USA) and 50 μg/mL PI (Sigma) in a dark box for 30 min at room temperature. The percentage of cells in different phases of the cell cycle was calculated by the Cell-QuestTM Pro software (BD Biosciences).

### Migration and invasion assays

The transwell migration and invasion assay was performed using standardized protocols as described earlier [9]. Briefly, 3 × 10^4^ cells in 100 μl serum-free media were added to the upper chamber of an insert (8- μm pore size, millepore) coated with or without Matrigel (BD). 600 μl media containing 10% FBS were added to the lower chamber. Cells were fixed and stained with 0.05% crystal violet after 12 hours or 36 hours for the migration and invasion assays, respectively. Six random fields of each chamber were photographed using an IX71 inverted microscope (Olympus, Tokyo, Japan) at 200× magnification.

### Western blot

Cells were harvested with 1× cell lysis buffer (Promega). A total of 60 μg of total proteins were separated on 10% polyacrylamide gel and transferred to nitrocellulose membranes (Bio-Rad). The membranes were blocked with 1% bovine serum albumin (BSA) in TBST buffer (Tris Buffer Saline containing 0.1% Tween-20) for 1 h at room temperature, and subsequently incubated with antibodies against FOXA1, BCL-2, CyclinD1 (Proteintech) or GAPDH (#2118, Cell Signaling Technology) overnight at 4°C. After extensive washing with TBST buffer, the blots were then incubated with goat anti-rabbit, horseradish peroxidase-conjugated secondary antibody for 1 h at room temperature. Protein bands were detected by enhanced chemiluminescence reagents ECL (Millipore, MA, USA) and the intensity of the bands was analyzed using Image J software (National Institute of Health, USA).

### Animal model

Six-week-old male nude BALB/c mice were purchased from the Shanghai Laboratory Animal Center of the Chinese Academy of Sciences (Shanghai, China). All mice were housed in specific pathogen-free conditions following the guidelines set forth by the Ethics Committee of the Shanghai Tenth People's Hospital of Tongji University. All protocols were approved by the Animal Care and Use Committee and by the local ethics committee of Tongji University.

To produce experimental tumour formation assay, A549 cells (1 × 10^7^) infected with lentiviral constructs carrying either the miR-194 vector or the control vector were harvested and were washed and re-suspended in serum-free RPMI-1640 medium, then were injected subcutaneously into the left flanks of anesthetized 4-week-old nude mice. Tumor growth was monitored by measuring the largest (a) and smallest (b) two perpendicular diameters with a caliper, and calculating the tumor volume (V) = a × b^2^ × 0.5. Tumor weights were also measured.

To produce experimental metastasis, A549 cells (1 × 10^6^) infected with lentiviral constructs carrying either the miR-194 vector or the control vector were harvested and injected into the 6-week-old nude mice. Mouse lungs were fixed with 10% neutral-buffered formalin, embedded in paraffin, sectioned at 4 μm and stained with H & E. The number of pulmonary metastatic tumor nodules was counted under a light microscope at magnifications of ×100 and ×200. All the mice were sacrificed at 5 weeks later.

### Statistical analysis

Data are presented as the mean ± SD (standard deviation). Student's *t*-test (two-tailed) or one-way analysis of variance was performed to analyze the *in vitro* and *in vivo* data. The association between miR- 194 expression and clinicopathological parameters was assessed using the Pearson Chi-Square test. Overall survival was calculated by the Kaplan-Meier method and evaluated by the log-rank test. All statistical analyses were performed using the SPSS version 16.0 software package (SPSS Inc., Chicago, IL, USA). A *p*-value < 0.05 was considered to be statistically significant.
